# Complex Deep Neural Networks from Large Scale Virtual IMU Data for Effective Human Activity Recognition Using Wearables

**DOI:** 10.3390/s21248337

**Published:** 2021-12-13

**Authors:** Hyeokhyen Kwon, Gregory D. Abowd, Thomas Plötz

**Affiliations:** 1 School of Interactive Computing, Georgia Institute of Technology, Atlanta, GA 30332, USA; hyeokhyen@gatech.edu (H.K.); g.abowd@northeastern.edu (G.D.A.); 2 Electrical and Computer Engineering, Northeastern University, Boston, MA 02115, USA

**Keywords:** human activity recognition, virtual IMU data, deep learning

## Abstract

Supervised training of human activity recognition (HAR) systems based on body-worn inertial measurement units (IMUs) is often constrained by the typically rather small amounts of labeled sample data. Systems like IMUTube have been introduced that employ cross-modality transfer approaches to convert videos of activities of interest into virtual IMU data. We demonstrate for the first time how such large-scale virtual IMU datasets can be used to train HAR systems that are substantially more complex than the state-of-the-art. Complexity is thereby represented by the number of model parameters that can be trained robustly. Our models contain components that are dedicated to capture the essentials of IMU data as they are of relevance for activity recognition, which increased the number of trainable parameters by a factor of 1100 compared to state-of-the-art model architectures. We evaluate the new model architecture on the challenging task of analyzing free-weight gym exercises, specifically on classifying 13 dumbbell execises. We have collected around 41 h of virtual IMU data using IMUTube from exercise videos available from YouTube. The proposed model is trained with the large amount of virtual IMU data and calibrated with a mere 36 min of real IMU data. The trained model was evaluated on a real IMU dataset and we demonstrate the substantial performance improvements of 20% absolute F1 score compared to the state-of-the-art convolutional models in HAR.

## 1. Introduction

Human activity recognition based on wearable sensing platforms (HAR) is a core component of mobile, ubiquitous, and wearable computing. Miniaturized inertial measurement units (IMUs), integrated into either body-worn devices such as smart watches, fitness bands, or head-worn units, or mobile devices such as smart phones are used to capture a person’s movements. These movement signals are then automatically analyzed to recognize and assess activities that are of relevance for many practical applications, including, for example, gesture-based interaction [1], health assessments [2,3], or behavioral authentication [4]. The algorithmic backbone for the vast majority of HAR techniques is based on machine learning, typically utilizing supervised learning methods to derive probabilistic recognizers that are used to segment and classify activities of interest [5].

Over the years, the HAR research community has developed numerous approaches to derive effective recognition systems [6,7,8,9]. Arguably, the biggest challenge and restricting factor for all supervised HAR learning methods is the often limited amount of labeled training data. Unlike in other application domains of machine learning, here it is challenging to collect large amounts of correctly annotated data samples. Reasons for these challenges are largely related to pragmatics of data collection procedures; it is simply impractical or even inappropriate to annotate real-life data, for example, by continuously following a person over extended periods of time. Likewise, it is impractical to ask a person directly to provide ground truth annotation for their everyday activities. As a consequence, many HAR datasets have been recorded in lab environments resulting in relatively small-scale datasets that do not always capture the richness of real-life activities.

The limited size of labeled training data for supervised model training is hindering progress towards more accurate activity recognition and towards analyzing more complex human activities, such as complex gestures and behaviors. To tackle this “small data” problem, the research community has explored many different avenues, ranging from, for example, data augmentation [10,11], transfer learning [12], to self-supervised learning techniques [13]. Recently, a paradigm change to data collection for supervised recognizer training has been introduced where existing repositories of videos of human activities are utilized to generate virtual IMU training data [14,15,16,17]. The key idea here is appealing—large numbers of videos capturing activities of interest can easily be retrieved from platforms such as YouTube. Through a computer vision-based processing pipeline, these videos can be assessed regarding their overall quality and suitability for the modality transfer into virtual IMU data. As a result, virtually unlimited amounts of (weakly) labeled training data can be generated automatically, serving as a basis for deriving robust activity recognition systems. Training datasets generated through systems such as IMUTube [14] can be used for significantly improved classification performance in HAR, thereby pushing the limits of conventional machine learning techniques, such as random forests, as well as of contemporary deep neural networks, such as DeepConvLSTM [7], arguably the de-facto standard in the field.

The architectures of state-of-the-art HAR models, such as the DeepConvLSTM model [7], were designed specifically respecting the restrictions of limited size labeled training sets. The popular DeepConvLSTM model [7] can only afford four, moderately-sized convolution layers to learn effective data representations, and two LSTM layers for sequence modeling. Attempts to increase the complexity of such models for scenarios based on current benchmark datasets of labeled training data very quickly lead to overfitting. With the effective alleviation of the “small data” problem as shown by systems like IMUTube, we are now in a position to rethink HAR modeling and to design more complex model architectures that are not primarily driven by limitations of the training data but rather can capture the characteristics of sensor data directly. In this work, we aim to demonstrate that HAR models with significantly increased model complexity compared to the popular DeepConvLSTM model can be effectively trained by making use of IMUTube for generating large-scale virtual IMU data. We hypothesize that with increased model complexity, i.e., more parameters trained robustly on labeled virtual IMU data, the overall accuracy of human activity recognition shall improve.

For the first time, in this paper we utilize a large-scale training set of virtual IMU data from IMUTube and video repositories to train more complex HAR models and demonstrate how these new models lead to substantially improved recognition accuracy. We automatically extracted training data from videos that were retrieved from YouTube through simple search queries with the search terms serving as (weak) labels for the supervised training procedure. We used the most recent version of IMUTube [15] to translate these videos into virtual IMU data. The design of the more complex HAR model is strictly oriented on the characteristics of IMU data and not restricted by potential limitations of small labeled training sets. Compared to state-of-the-art model architectures, we increase the number of trainable parameters by a factor of 1100. We demonstrate the effectiveness of the new model architecture on a challenging application case, namely the automated, fine-grained assessment of free weight gym exercises. Our models lead to a gain of more than 20% absolute in the F1 score compared to the state-of-the-art convolution models. Such a significant improvement confirms our assumption that more complex models lead to improved recognition performance—if trained robustly. We show that systems like IMUTube lead to an effective elimination of restrictions on model training imposed by small-size training sets that allow for the aforementioned robust training of more complex models.

## 2. Background

Machine learning plays a central role for most activity recognition scenarios. Traditionally, (variants of) the activity recognition chain (ARC, [5]) have been employed. While ARC-based methods are still of value for various application scenarios, deep learning-based methods have now taken over the field. However, for many application scenarios, such sophisticated models are difficult to derive in a robust way. The labeled datasets, that are needed for supervised model training, are simply too small—a challenge we refer to as the “small data(set)” problem. In what follows, we will first give a brief overview of relevant modeling techniques before we summarize how the community is addressing the “small data” problem. We end with a description of the specific IMUTube tool that is used in this paper.

### 2.1. Human Activity Recognition Using Wearables and Machine Learning (HAR)

#### 2.1.1. Conventional Modeling through the Activity Recognition Chain

A traditional activity recognition system typically implements (variants of) the activity recognition chain (ARC [5]). Data is recorded, pre-processed, and segmented into individual analysis frames that contain a fixed number of consecutive sensor readings. For these frames features are extracted, which are then used for classifier training (and evaluation).

Building activity recognition system starts with data collection from users. In such data recording sessions, users are invited to a laboratory environment, and data is recorded and annotated as users perform sets of activities. The dataset is then preprocessed to remove noise and to normalize the sensor readings with regards to range, sampling rate, synchronization, etc. [18]. In order to automatically analyze sensor data, the recording stream is divided up into individual frames using a sliding window procedure. For each analysis window, features are calculated, often based on hand-crafted representations that incorporate statistical, frequency, or distribution information [19,20,21]. As the final step of the processing pipeline, machine learning models, such as support vector machines or random forest, are trained as classification backend.

#### 2.1.2. Feature Learning

As an alternative to the often not generalizable hand-crafted feature representations, the HAR community has been actively exploring the automated extraction of data representations through feature learning thereby often employing deep learning methods [6,22]. Plötz et al. [6] demonstrated that feature learning using deep belief networks (DBN) can lead to significantly improved and especially generalized classification performance downstream when compared to hand-crafted features. Other variants of feature learning utilized more contemporary forms of autoencoder networks showing similar improvements in feature generalization capabilities [23,24].

Recently, end-to-end learning approaches have been introduced for supervised feature learning in HAR [22,25]. Such methods typically employ deep neural networks and train both the feature extractor and classifier parts of an integrated network simultaneously [11,26]. In order to capture the temporal structure of the sensor time-series, Ordóñez and Roggen proposed a hybrid model that combines both convolutional and recurrent neural networks [7].

Convolutional neural networks learn feature extraction kernels that capture local temporal patterns, which are then aggregated through multiple layers and in a hierarchical manner for effective data abstraction. Combined with recurrent neural network elements such as long short-term memory (LSTM) cells [27], effective models can be derived that capture both short- and long-term temporal dependencies by aggregating historical features along the sequence [7,25,26,28]. Feature representations from such models are then processed with multiple layers of fully connected models (multi-layer perceptron) using either sigmoid or softmax activation functions for binary or multi-class classification tasks, respectively.

### 2.2. Tackling the Small Data Problem in HAR

Collecting large-scale sensor data for human activity recognition is challenging when targeting real-life situations. Typically, such endeavors have to be conducted in laboratory environments to allow for manual data annotation. Since this process is expensive, time-consuming, and labor-intensive, typically only relative small amounts of labeled data can be collected—often covering only a few dozen users and activities, performed over relatively short periods. For example, the Daphnet freeze of gait dataset consists of a mere five hours of sensor data from 10 users [29], and the Wetlab activity dataset covers 13 hours of sensor data from 22 users [30]. Moreover, human activity datasets commonly exhibit class imbalance with the NULL class being over-represented. A dataset such as Opportunity has 75% of the samples assigned to the NULL (or ‘other’) class [31], which makes it difficult to build classifiers without overfitting. Class imbalance in training datasets poses challenges for designing classifiers not to neglect minority class samples. In the field of human activity recognition, there have been multiple ways proposed to address this ‘small data(set)’ problem, including data augmentation, transfer learning (or domain adaptation), self-supervised learning, and cross-modality adaptation. We provide brief descriptions of each category of approaches below.

#### 2.2.1. Data Augmentation

Using data augmentation techniques, artificial data samples are generated from the original data by adding noise or by applying specific geometric transformations. Um et al. [11] improved their freeze-of-gait detection model by applying permutations, rotations, and time warping operations on the original training set. Le et al. [10] and Fawaz et al. [32] applied data warping techniques, including window slicing, window warping, rotations, permutations, and dynamic time warping to existing datasets to augment their training base. Fernández et al. [33] also proposed techniques to oversample minority samples to tackle the label imbalance problem.

Alternatively, generative model-based processes have been used to extend the size of training datasets in HAR. In particular, generative adversarial networks (GANs) have been adopted [34,35]. For example, Harada et al. [8] and Yao et al. [36] augmented biosignals and IoT (Internet of Things) sensor signals with GANs, respectively. Ramponi et al. [37] extended conditional GANs to augment irregularly sampled time-series data. Despite outperforming data transformation approaches, GAN-based models showed only modest performance gains, probably due to the difficulty of generating realistic time series data.

#### 2.2.2. Transfer Learning and Self-Supervised Learning

A large body of research has explored transfer learning methods to tackle the small data problem [12,26]. Transfer learning first trains a classifier on a base dataset/task. Then, the trained classifier is transferred to the target task by re-purposing (or finetuning) the representations (features) learned on the original task. For cases where the target task came with only small datasets (compared to the base dataset for pretraining the original model), transfer learning could significantly improve the classification performance [38]. Gjoreski et al. [9] demonstrated that successful transfer learning depends on the similarity of the domain between the base and target task. Hu et al. [39] proposed an unsupervised technique to select the base task most effective for the target downstream task. Chen et al. [40] developed an online domain adaptation model under the challenge of dynamically changing feature dimensions, activity classes, and data distributions, simultaneously.

Recently, self-supervised learning has gained popularity in HAR as a form of transfer learning. A self-supervised learning approach pre-trains a model using a “pretext” task, rather than a base dataset. The “pretext” task is a prediction task derived with domain expertise to provide supervisory signals related to downstream analysis tasks such as activity recognition. The pretrained model is then finetuned with a small dataset from target task. Saeed et al. [41] proposed a multi-task pretext task to predict eight different types of data transformations applied to the input data. Haresamudram et al. [13] pretrained a transformer network [42] to reconstruct signals in randomly masked timesteps in sensor frames. For a federated learning framework involving multiple sensors, such as WiFi, IMU, electroencephalogram, and blood volume pulse, Saeed et al. [43] applied contrastive learning with wavelet transformations. Combining contrastive learning with predictive coding, Haresamudram et al. [44] showed that the performance of downstream classification tasks could be significantly improved.

#### 2.2.3. Cross-Modality Transfer

Transfer learning and data augmentation help mitigate the “small data” problem by utilizing sensor datasets, i.e., data of the same original modality. Research is also underway to train HAR models using data from other modalities, including character animations, motion captures, and human activity videos. Kang et al. [45] extracted IMU signals from motion information generated by hand-designed 3D character animations using the Unity game engine [46]. Designing and simulating complex human activities is very challenging such that their dataset is limited to simple gestures and locomotions. However, the usefulness of virtual IMU data from virtual 3D characters has inspired other studies to explore large, public motion capture datasets available, for example, in the computer graphics community [47,48,49]. Xiao et al. [50] and Takeda et al. [51] trained HAR models with virtual IMU data extracted from hundreds of subjects performing thousands of motions in the motion capture dataset. The training of HAR models with large-scale virtual IMU datasets resulted in a significant improvement in classification performance. Nevertheless, motion capture datasets are typically limited in capturing the full range of everyday human activities.

Video repositories have recently attracted attention as potential training resources for sensor-based HAR models [14,15,17]. Sites like YouTube host vast amounts of videos that capture broad ranges of human activities in real-world scenarios. Utilizing computer vision techniques has allowed researchers to extract core motion information about a person’s activities in a video and then to translate these into virtual sensor data. As an example, Rey et al. [17] regressed 2D keypoint location changes resulting from a person performing activities in a video into time-synced accelerometer data. Kwon et al. [14,15] estimated full 3D motions of multiple people in the video and derived 3D virtual IMU data from the body part where the sensor was, virtually, attached. The IMUTube system serves as the basis for the work presented in this paper, and a more detailed overview of the system is given below.

### 2.3. Generating Large Scale Virtual IMU Data from Real World Videos Using IMUTube

**Figure 1:**
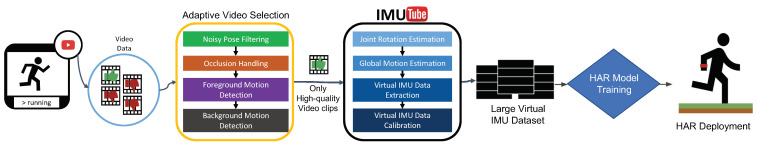
Deriving human activity recognition systems from large scale virtual IMU data generated through IMUTube [14]. Videos for specific activities are retrieved from public repositories such as YouTube through keyword-based queries. These search terms serve as weak labels for all generated sensor data. The IMUTube system then processes the retrieved videos to *(i)* suppress irrelevant videos; *(ii)* eliminate portions of the videos that are of insufficient quality (motion blur, occlusions, etc.); *(iii)* generates virtual IMU data for on-body sensor positions as specified by the query; and *(iv)* calibrates the virtual IMU data towards more realistic sensor data. The resulting, large-scale virtual IMU dataset is then used for HAR model training.

IMUTube was introduced as a video retrieval and processing system that allows to make use of large-scale video datasets for training IMU-based activity recognition models [14,15]. IMUTube generates virtual IMU data from videos that have been retrieved from online repositories such as YouTube by querying these for activities of interests. The search terms, such as “cycling” or “biceps curl”, serve as (weak) label for the virtual IMU data that are generated using IMUTube. The overall assumption for the cross-modality adaptation is that the motion signals that are relevant for individual activities are captured by both the video cameras and the (virtual) IMU sensors. IMUTube bridges the gap between the two modalities by effectively transferring videos into IMU data through a fully automated procedure. Figure 1 gives an overview of the IMUTube principle for generating virtual IMU training data from videos.

To collect high-quality, realistic yet virtual IMU data, accurate human motion information needs to be extracted via 3D human motion tracking from unconstrained 2D videos. To do so, IMUTube implements a processing pipeline that incorporates techniques from the computer vision, computer graphics, and signal processing domains. Unconstrained videos of human activities as they can be found in repositories such as YouTube often stem from non-professionals who may use handheld cameras in non-ideal recording conditions. These conditions result in multiple challenges for the automated process that aims at extracting human motion information, including motion blur, occlusions, and extreme camera movements to name but a few. It is not yet possible to track 3D motion accurately under the presence of such artifacts using state-of-the-art computer vision techniques. IMUTube tackles these challenges by automatically filtering out videos and segments thereof that fall short in recording quality, which would result in poor quality virtual IMU data. As such, the IMUTube processing pipeline selectively chooses videos that are most suitable for conversion into virtual IMU data. Given the sheer size of public video repositories, such lossy data collection is appropriate.

#### 2.3.1. Adaptive Video Selection

Given a set of human activity videos that are returned by a query process that is focused on the textual description of the activity of interest through appropriate search terms (“cycling, or ”biceps curl”), IMUTube first detects and removes video segments that contain certain artifacts, which may lead to a degradation of the quality of human motion tracking results. Examples of such artifacts include noisy human poses, occlusions/self-occlusions, and (too) fast foreground/background motions (Figure 1, orange box).

**Noisy Pose Filtering:** For motion tracking, IMUTube detects 2D poses in a video sequence. Standard multi-person 2D pose detectors often confuse people in a scene with non-human objects that may look like humans, which results in erroneous tracking [52]. To avoid collecting such faulty motion data from non-human objects, IMUTube first employs a human detector [53] to automatically find humans in a video frame. The system selects the detected human with the highest confidence score to track and estimate 3D motion through the remaining operations of the overall processing pipeline.

**Occlusion Handling:** The presence of occlusions in a 2D video can also complicate the tracking of human poses and motion. As a result of occlusions between people and between people and objects in a scene, and self-occlusions, i.e., the overlap of body parts of individuals, human pose detectors often struggle determining where key points of the to-be extracted skeleton model should be placed. IMUTube detects potential occlusions for the position the virtual sensor shall be placed on and excludes those portions of a video from further processing. To do so, the system applies human segmentation [54] and body part parsing [55] for a detected human bounding box. The human segmentation mask is compared with the locations of detected body keypoints to determine if the body keypoint is occluded or not. If the body keypoint location is outside of the segmentation mask, it is flagged as occlusion and excluded from further processing. For those video portions with non-occluded human poses, self-occlusion is examined next. IMUTube compares the location of the body part keypoint with the body part parsing results. Keypoints detected in the wrong part of the body, such as wrist keypoints in the lower body parts if the virtual sensor shall be placed at the wrist, will result in the pose being deemed self-occluding and removed from further processing.

**Extreme Foreground & Background Motion Detection:** All remaining human poses and video segments are then examined for potential extreme background or foreground movements. A person’s fast movements can cause motion blur in the video footage, which may result in erroneous pose estimation such as estimating multiple poses from a single individual. IMUTube detects such spurious poses by tracking changes in keypoint locations and human bounding box shapes between successive frames. When extreme value changes are detected, IMUTube discards such poses from further processing. Moreover, rapid camera movements can produce extremely blurry background images that pose significant challenges in estimating human motion. IMUTube quantifies background motion by using optical flow estimations [56]. Optical flow measures displacements of each pixel between subsequent frames. Background motion is characterized by the intensity of background optical flows accumulated from the non-human area of the frame. Those frames that exhibit extremely large background motion are removed from further analysis.

#### 2.3.2. 3D Human Motion Tracking and Virtual IMU Data Extraction

The adaptive video selection procedure described above reduces the retrieved videos for target activities to those that contain video frames that can be used for extracting high-quality virtual IMU data. Subsequently, the system analyzes these remaining video segments with regards to tracking 3D human motions through generating joint and global motion estimations from which virtual IMU data can be extracted and calibrated for model training (Figure 1, black box).

**Joint Rotation Estimation:** IMUTube first identifies the rotation of each joint in a video. To do so, the system estimates 2D poses [52,57] from detected humans using a human detector [53]. In order to estimate 2D pose changes across video sequences, a multi-person tracker is utilized [58,59]. Then each 2D pose sequence is lifted to 3D poses using 3D pose estimators [60]. For capturing temporally smooth rotations of 3D joints, Kalman filters are applied to the detected 3D pose sequences.

**Global Motion Estimation:** Videos of human activities may be recorded through the camera following the person who is engaging in the target activity, resulting in substantial camera movements that are not of relevance for the analysis of the target activity and may actually degrade the quality of the generated data. IMUTube needs to estimate global body acceleration across the entire video sequence while compensating for camera movements in order to extract high-quality virtual IMU data. The body locations and orientations of each tracked person within each frame are estimated by calibrating previously estimated 3D human poses with the projective relationship for the matched 2D human poses in the scene using Projective-n-Point estimation [61]. In the next step, IMUTube calculates the camera ego-motion to account for 3D human pose location and orientation in accordance with the camera movements. IMUTube does so by estimating the background depth maps from each scene and lifting them to 3D point cloud models [62,63]. Based on subsequent 3D point clouds, IMUTube calculates the camera’s ego-motion using the Iterative Closest Points (ICP) method [64]. Finally, the camera ego-motion-compensated 3D global motion is integrated with previously estimated 3D joint rotations throughout the entire video sequence.

**Virtual IMU Data Extraction and Calibration:** Following the estimation of the full 3D motion for each person in the video, IMUTube extracts virtual IMU data from specific body locations of interest. Based on forward kinematics from a predefined body center, i.e., the hip, the motion of the target body locations and orientation can be tracked, which results in estimates of linear accelerations and rotational velocities for local sensor coordinates [65]. To do so, IMUTube uses the IMUSim model to generate realistic virtual IMU data [66]. IMUSim simulates noise from mechanical and electronic components in real IMU sensors. The extracted virtual IMU data is then calibrated for the real IMU dataset that is used during deployment. The specifications of different IMU sensors may vary with regards to, for example, drift noise, bias, and the range of output values. In order to minimize the discrepancies between the extracted virtual IMU data and the target real IMU sensor data, IMUTube applies distribution mapping [67].

Finally, the calibrated, virtual sensor data are used for training HAR models. Search queries serve as (weak) ground truth annotation of the training data. The advancements in IMUTube have made it easier to collect weakly-labeled virtual IMU datasets, which have been successfully used for exemplary HAR tasks, including classification of locomotions, home activities, and gym exercises [31,68,69].

## 3. Complex Deep Neural Networks for Human Activity Recognition

With the availability of large-scale, annotated training sets of (virtual) IMU data we are now in the position to freely design complex model architectures for deep learning-based HAR. Large training sets us free to focus on capturing the relevant characteristics of IMU data with models that have larger numbers of trainable parameters. The more complex models result in improved activity recognition performance. In what follows, we first give an overview of the novel model architecture before we give detailed explanations of all model components.

### 3.1. Model Overview

**Figure 2:**
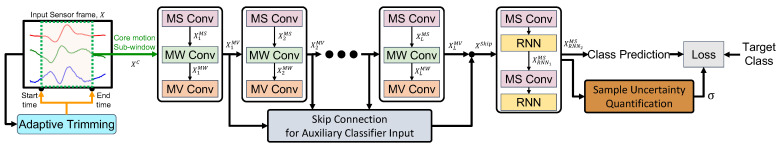
Overview of the novel model architecture for deep learning-based human activity recognition using wearables. An input sensor frame is first processed with Adaptive Trimming (AdapTrimm or AT) to determine the sub-window region that contains the core motion of the target activity. The feature representation is then extracted for this core motion sub-window (green segment). Non-linear multi-scale convolution (MS Conv) captures temporal dynamics at multiple temporal scales. Multi-kernel window convolution (MW Conv) captures varying lengths of motion signals from multi-scale features. Multi-view convolution (MV Conv) uses diverse shapes of convolutional kernels to capture time-channel correlations. The feature representation from intermediate convolutional layers is used as input to the final classifier in the form of auxiliary inputs with skip-connections to enhance gradient flow in the bottom layers of the recognition network. The convolutional feature representations are aggregated by a non-linear multi-scale recurrent neural network (MS RNN), which is used to predict the activity class of the input sensor frame. Along with the class label, the confidence for the prediction is generated by the model, which is for loss calculation to regularize the contribution of noisy inputs for model updates during training.

In this work, sensor data is–in general–processed by following the standard deep learning-based HAR paradigm [7,25]. A small analysis window is slid along the sensor data stream and the resulting frames form the basis for all subsequent processing. Each sensor frame is forwarded to the complex analysis model that we introduce in this paper, which is trained in a supervised manner by utilizing the search queries as activity labels for the extracted sensor frames using standard backpropagation [70]. Figure 2 gives an overview of our new model architecture that starts off with the input sensor data frames extracted through the aforementioned sliding window procedure. The new analysis model targets improvements of the following three core parts of sensor data analysis: *(i)* Sensor stream segmentation; *(ii)* sensor feature representation; and *(iii)* model training. We introduce adaptive sensor window trimming that exploits automated detection of core motion sequences, novel convolutional neural network models for effective feature learning, and sample uncertainty quantification for handling noisy labeled samples. Specifically, the adaptive trimming model (Section 3.2) captures that segment in a window of sensor data that is most relevant for a given activity recognition task (core motions). Analysis windows extracted by the sliding window method and curated through adaptive trimming are then processed to extract multi-scale (Section 3.3.1), multi-kernel window (Section 3.3.2), and multi-view (Section 3.3.3) feature representation to capture multiple temporal scales and varying lengths of human actions. The HAR model is trained through adaptive learning that explicitly takes into account potential sample uncertainty such that descriptive and discriminative feature representations can be learned from raw, virtual IMU data (Section 3.5).

### 3.2. Adaptive Trimming of Sensor Window for Detecting Core Motion Signal

Following the standard sliding-window paradigm, we first segment continuous streams of sensor data. The window size is an important design factor for recognition performance. It has to be large enough to capture the core motion in an ongoing activity while at the same time short enough to exclude irrelevant motion parts. In contrast to previous work, our method does not use a fixed analysis window but rather explicitly learns a core motion detector, which can adaptively capture the key aspects of the signal as they are of relevance for target activities.

As shown in Figure 2, our Adaptive Trimming (AdapTrimm or AT) model automatically detects the start and end times of core motions as defined above. Given a sensor frame $X\in R^{T\times d}$ of length *T* and dimension *d* (number of channels) that is sufficiently large to capture target activities in various contexts, AdapTrimm first predicts the center location *c* (index in the data stream) and width *w* (number of samples to be included in the window) of the sub-window that contains the core motion:

(1) $$\begin{array}{cc} c & =sigmoid(F^{center}\left(F_{at}\left(X\right)\right)) \end{array}$$

(2) $$\begin{array}{cc} w & =\exp(F^{width}\left(F_{at}\left(X\right)\right)). \end{array}$$

In a given sensor window, $F_{at}\left(x\right)$ extracts the features using a four-layer convolutional model for regressing two real values corresponding to the center and width of the core motion signal. Based on the extracted features, $F^{center}$ and $F^{width}$ are two-layered fully connected models for predicting the center 0<c<1 and the width w>0.

Following this, the center location *c* and window width *w* are used to derive start *s*, and end *e* indices of the core sub-window, where 0<s,e<T:

(3) $$\begin{array}{cc} s & =T\times sigmoid(c-\frac{w}{2}) \end{array}$$

(4) $$\begin{array}{cc} e & =T\times sigmoid(c+\frac{w}{2}) \end{array}$$

(5) $$\begin{array}{cc} X^{C} & =X\left[s:e\right]=F_{crop}\left(X,s,e\right). \end{array}$$

The core sub-window $X^{C}\in R^{T^{\prime }\times d}$, where $T^{\prime }=e-s$, is passed on to the feature extraction part of our model. If the fixed-size input is needed for the subsequent classifier, the cropped sub-window can be either interpolated or zero-padded to the required size.

The cropping operation, $F_{crop}\left(X,s,e\right)=F_{sampler}\left(F_{grid\_gen}\left(X\right),s,e\right)$, of AdapTrimm is fully differentiable, which is adapted from the grid generator, $\overset{´}{X}=F_{grid\_gen}\left(X\right)$, and sampler, $F_{sampler}\left(\overset{´}{X},s,e\right)$, used in spatial transformer networks (STN) [71]. STNs learn to adaptively apply geometric manipulations, such as translation, scaling, or rotation to given input data to localize their most relevant parts. For the geometric manipulations, STNs first generate a parameterized 2D affine grid for the location of the relevant parts of the data and then samples the grid to generate new data samples that only contain the target parts. The grid generator and sampler are fully differentiable operations, which effectively resemble an interpolation process. For AdapTrimm to crop the input sensor stream, the 1D temporal grid is generated from the detected start and end time of the core motion signal, and the core motion segment is sampled according to the generated 1D grid.

### 3.3. Multi-Scale, Multi-Window, Multi-View Convolutional Model

Once the core motion parts have been determined by AdapTrimm, as described in the previous section, our model focuses on extracting three different kinds of features that capture the essentials of the underlying movement data, specifically targeting key information that is of relevance for a subsequent classification task: *(i)* Multi-scale temporal information; *(ii)* multiple lengths of motion units; and *(iii)* time-channel correlations. In what follows we provide the technical details of this feature extraction process that is integrated into our end-to-end learning procedure.

#### 3.3.1. Non-Linear Multi-Scale Feature Representation

Different frequency components of a signal represent different levels of motion information. Typically, global and dynamic acceleration information is captured by low- and high-frequency components, respectively. To incorporate such multi-scale motion information into our data representation, we capture sensor features at multiple temporal scales of the underlying input time-series (MS-Conv) by using multiple branches of hourglass network models [72,73,74]. Hourglass network models are often used for, e.g., image segmentation or human pose estimation tasks. Such networks are deemed effective for capturing low-dimensional spatial representations of images. Aiming to directly capture the relevant frequencies in the (trimmed) signales, we adopt hourglass models such that each model targets different scaling factors of the input data [72].

**Figure 3:**
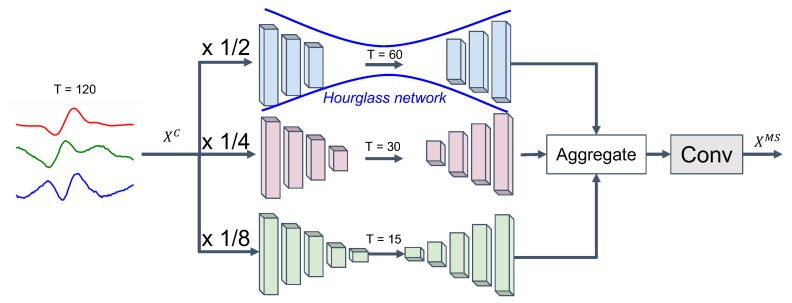
Non-linear multi-scale feature extraction (MS-Conv) with rescaling factors of $\left[\frac{1}{2},\frac{1}{4},\frac{1}{8}\right]$. To capture low dimension temporal representation at each scaling factor, we adopt hourglass networks, which are often used to capture low dimensional spatial representations of 2D images in computer vision scenarios [72]. The hourglass network first downsamples input with the convolutional model and then upsamples back to the original scale with the transposed convolutional model with ReLU activation. We apply separate hourglass network models to capture each temporal scale information independently, which is then aggregated and remixed with the bottleneck convolutional layer for final output.

Input segments are first downsampled through a temporal convolution (left part of models in Figure 3) and then, after passing through the intentional bottleneck (middle part of models in Figure 3), upsampled by the transposed temporal convolutional model (right part of models in Figure 3). Assume $\left[\frac{1}{2},\frac{1}{4},\frac{1}{8}\right]$ are defined as the range for the temporal scales with three branches in our hourglass network model from $X^{C}\in R^{h\times T\times d}$, where *h* is the number of feature map, *T* is the temporal length, and *d* is the number of sensor modalities (Figure 3). Then, the targeted multi-scale feature representation, $X^{MS}=F_{ms}\left(X^{C}\right)$, is extracted from the MS-Conv model:

(6)XMS=Fms(XC)(7)=F5×1(concath([U×2(D×12(XC)),U×4(D×14(XC)),U×8(D×18(XC))]))

$U_{\times m}$ and $D_{\times \frac{1}{m}}$ are non-linear up- and downsampling operators with factor *m*, defining multiple layers of the transposed and the non-transposed convolutional models, respectively. We use a kernels size of 5×1 and a stride size of 2 with ReLU activation for both convolution and transposed convolution. The outputs of all rescaling branches are concatenated in a feature map and the bottleneck layer, $F_{k_{1}\times k_{2}}\left(\cdot \right)=ReLU\left(conv_{k_{1}\times k_{2}}\left(\cdot \right)\right)$, is used to aggregate multi-scale feature representation from each branch. For the bottleneck layer, we use a temporal convolutional operator with 5×1 kernel.

#### 3.3.2. Multiple Kernel Window Size for Capturing Varying Motion Length

For different human activities across different people, the length of the core motion signal may differ. For example, the average duration of a (walking) step is between 0.3 and 0.6 s depending on age [75,76,77]. Depending on the specifics of eccentric motions in, e.g., gym exercises (as we study them in this paper) these durations can get extended to 1–3 s [78,79]. Modeling such variations of motion lengths is important for deriving effective feature representations, be it explicitly through feature engineering or implicitly through end-to-end learning [80,81]. Our model explicitly captures varying lengths of movement signals by utilizing multi-length kernel windows (multi-window) for the convolution operation, i.e., feature extraction.

In our multi-kernel window convolution (MW-Conv) method we use a variety of kernels with sizes ranging from 3×1 to 90×1 sensor readings. For an exemplary 30-Hz signal a 3×1 kernel extracts 0.125 s of motion signal, whereas the 90×1 kernel covers 3 s of input data. Given a feature output from the preceding MS-Conv model, $X^{MS}=F_{ms}\left(X^{C}\right)\in R^{h\times T\times d}$, we compute multi-window feature representation $X^{MW}=F_{mw}\left(X^{MS}\right)$ through our MW-Conv model:

(8) $$X^{MW}=F_{mw}\left(X^{MS}\right)=F_{5\times 1}\left(concat_{h}\left(\left[F_{3\times 1}\left(X^{MS}\right),\;F_{5\times 1}\left(X^{MS}\right),\;\cdots ,\;F_{91\times 1}\left(X^{MS}\right)\right]\right)\right).$$

**Figure 4:**
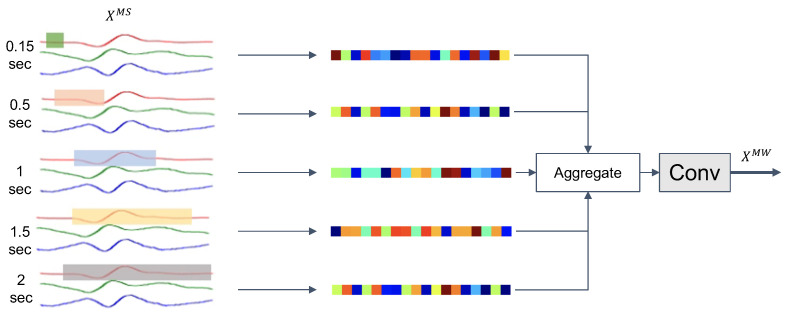
Multi-kernel window convolution (MW-Conv) with kernel sizes of [0.15,0.5,1,1.5,2] seconds, respectively, to capture short and long motions in human activities. We used different colors for each kernels to show that they are independent and not sharing the weights. Features from each kernel are concatenated along the feature map axis and recombined through the bottleneck layer. We have explored other feature aggregation approaches, such as summation or bilinear pooling [82], but concatenation was most effective over others.

The resulting features, extracted using the set of kernels with different sizes and shapes, of varying temporal kernel sizes are concatenated along the feature map axis. The concatenated feature vector is then passed through the bottleneck layer to recombine the multi-kernel window feature representation. Figure 4 illustrates the procedure.

#### 3.3.3. Multi-View Kernels for Time-Channel Representation

**Figure 5:**
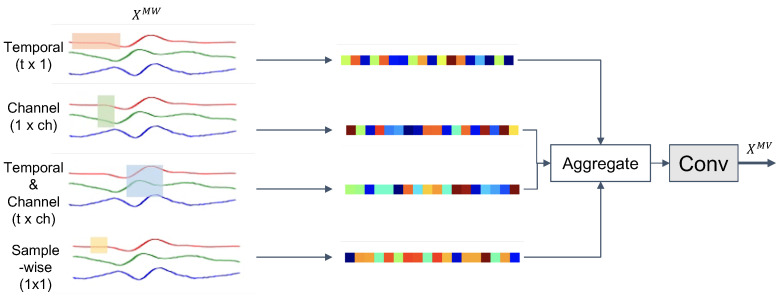
Multi-view convolution (MV-Conv) with kernel shapes t×1, 1×c, t×c, and 1×1 are used to capture the time and channel correlations. We used different colors for each kernels to show that they are independent and not sharing the weights. Features from each kernel are concatenated along the the feature map axis and recombined through the bottleneck layer. We have explored other feature aggregation approaches, such as summation or bilinear pooling [82], but concatenation was most effective over others.

For effective activity recognition, feature representations for multi-channel sensor data in HAR models need to capture the relationships between time and channel information [83]. In order to extract time-channel correlations from input time-series $X^{MW}\in R^{h\times T\times d}$ we process sensor data through four different views of convolutions (MV-Conv), as shown in Figure 5. Kernels of shapes t×1 and 1×c implement convolutions along the time and channel axes, respectively, to capture the features according to each view of the sensor time-series. The t×c kernel implements a 2D convolution along both axes to capture local correlations of time and channel patterns. We also apply a 1×1 kernel to extract sample-wise features that are independent of any time or channel axis. Subsequently, the four features extracted through the individual kernels are fused, resulting in an aggregated representation that effectively integrates information from four different viewpoints on a multi-modal sensor stream. Given a feature output from the preceding MW-Conv model, $X^{MW}=F_{mw}\left(X^{MS}\right)\in R^{h\times T\times d}$, the multi-view convolution MV-Conv with kernels of shape t×c is derived as $X^{MV}=F_{mv}^{t\times c}\left(X^{MW}\right)$ via:

(9)XMV=Fmvt×c(XMW)(10)=F5×1(concath([Ft×1(XMW),F1×c(XMW),Ft×c(XMW),F1×1(XMW))])).

The four feature sets are concatenated along the feature map axis, and a bottleneck layer is used to recombine the concatenated features.

### 3.4. Full Feature Extraction Model with Skip-Connection and Temporal Aggregation

So far we have introduced multi-scale (MS-Conv), multi-window (MW-Conv), and multi-view (MV-Conv) convolutional models to capture relevant aspects of sensor time-series. In what follows, we show how those convolutional modules are used in the overall feature extraction scheme. We also introduce skip connections to enhance gradient backpropagation for model training and incorporate recurrence into the overall network architecture to facilitate temporal aggregation of convolutional features for classification.

#### 3.4.1. Composite Convolutional Layer

The proposed model employs a sequence of multi-scale, multi-window, and multi-view convolution—each integrated into the MSMWMV-Conv layer. MS-Conv (Section 3.3.1) is first applied to the input time-series $X^{MS}=F_{ms}\left(X^{C}\right)$. Then the resulting multi-scale feature representations are further processed to facilitate multi-window (Section 3.3.2), multi-view (Section 3.3.3) convolution through MWMV-Conv. Multi-window multi-view convolutions (MWMV-Conv) capture time-channel correlated features at different motion length ranges. Specifically, MVMW-Conv feature representations $X^{MV}=F_{mwmv}\left(X^{MS}\right)$ are extracted as follows:

(11)XMV=Fmsmwmv(XC)=Fmwmv(Fms(XC))=Fmwmv(XMS)(12)=F5×1(concath([Fmv3×3(XMS),Fmv5×3(XMS),⋯,Fmv61×3(XMS)]))

where multi-view features are captured for different temporal lengths [3,4,⋯,61]. The extracted features are concatenated and aggregated with a bottleneck layer. Lastly, we use multiple MSMWMV-Conv layers to extract high-level sensor features.

#### 3.4.2. Handling Vanishing Gradients with Skip Connections

The basis for our novel model architecture are multiple stacks of MSMWMV-Conv layers to represent high-level feature representations extracted from the raw sensor input data. As MSMWMV-Conv layers are stacked, the width and depth of the model increase significantly. Compared to the standard convolutional network model, which uses a single convolutional kernel in each layer, the size of the proposed model in terms of model parameters has multiplied compared to standard models in the field (such as DeepConvLSTM [7]). The intended increase in the number of trainable model parameters stems from the increased number of kernels used for feature extraction, their sizes, and their shapes (as described before). For example, the number of convolution kernels for a single MSMWMV-Conv layer increased to 25 from a single kernel per layer in a standard convolution layer, with $\left[1,\frac{1}{2},\frac{1}{4}\right]$ scales, [5,11,15] kernel windows, and four different shapes for multi-view convolution.

**Figure 6:**
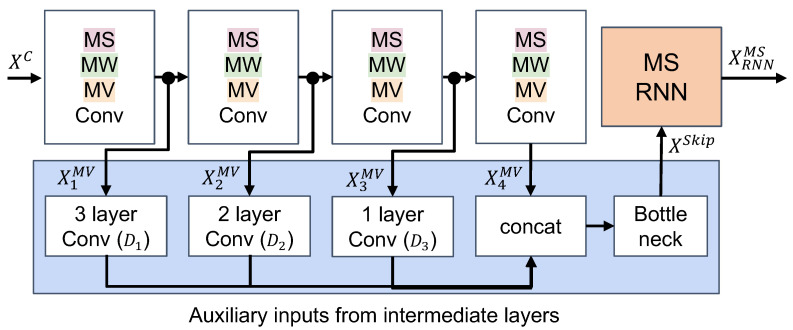
MSMVMW-Conv model with skip connections and temporal aggregation through multi-scale recurrent neural network. To handle vanishing gradients at the bottom layers of the MSMVMW-Conv model, skip connections are introduced to directly feed feature representations from intermediate layers as auxiliary inputs to the classifier (blue). In this example, feature representations from each intermediate layer are processed by the multi-layer convolutional neural network with max-pooling to match the size of the feature representation from the final layer. The fused features from all convolutional layers are passed to the multi-scale recurrent neural network model for temporal aggregation and classification (orange).

In our experiments, we use four MSMWMV-Conv layers for convolutional feature extraction. Previous work showed that training a large model is challenging because it becomes more likely that the gradient during optimization becomes infinitesimally small at the bottom layers [84,85,86]. The weights in multi-layer neural network models are updated during training proportional to the partial derivative of the error function with respect to the current weights. Backpropagation is calculated through the chain rule for derivatives. Multiple multiplications of small gradients in higher layers exponentially decreases the gradient error signals in the bottom layer effectively stopping the weight from further training. Adding so-called skip-connections from intermediate layers directly to layers later in the model seemingly helps to counteract the vanishing gradient by preserving the flow of backpropagated gradient signals [86,87]. As shown in Figure 6, we introduce skip-connections into our new, complex HAR model, specifically from all intermediate layers to the final layer (classifier input) through $F_{ax}\left(x\right)$:

(13)XSkip=Fax([X1MV,X2MV,⋯,XLMV])(14)=F5×1(concath([D1(X1MV),D2(X2MV),⋯,XLMV])).F5×1(concath([D1(X1MV),D2(X2MV),⋯,XLMV])).

Features $X_{l=1,\cdots ,L-1}^{MV}$ from intermediate MSMWMV-Conv layers l=1,⋯,L−1 are concatenated with the final output $X_{L}^{MV}$. When a downsampling operator, such as max-pooling, is used at each convolutional layer, the dimensionality of the feature vectors decreases along the hierarchy of the model. We apply multi-layer convolutional models with max-poolings, $D_{l}\left(\cdot \right)$, to downscale the feature representation from early model layers to match the size of the feature representation of the final layer. Similar to previous operations, the concatenated feature vectors are again processed through the bottleneck layer, and then fed to the classifier.

#### 3.4.3. Temporal Aggregation with Multi-Scale Recurrent Neural Network

Recurrent neural networks (RNNs) are often used in combination with convolutional operators in an effort to capture global temporal dynamics of the feature representations learned by the convolutional part of the model [7]. Furthermore, studies have shown that multi-scale recurrent neural networks are effective through hierarchically aggregating the inherent temporal dynamics of the data sequences analyzed [88,89,90].

Accordingly, we adopt and adapt multi-scale RNNs with multi-branch hourglass networks similar to what was described earlier (Section 3.3.1). First, the output of the final convolutional layer, $X^{Skip}\in R^{h\times t\times c}$, is rearranged to match the sequential input to the RNN: $x^{Skip}\in R^{t\times d}$, where d=h×c. Similar to what was described in Section 3.3.1, the input is then rescaled according to the desired scaling factors by using the temporal hourglass network. Given the feature representations $X^{Skip}=F_{ax}\left(X_{l=1,\cdots ,L}^{MV}\right)$ from the skip-connection layers, when using $\left[1,\frac{1}{2},\frac{1}{4}\right]$ as scaling factors, the multi-scale RNN feature representation $x_{RNN}^{MS}=F_{msrnn}\left(X^{Skip}\right)$ becomes:


(15)xRNNMS=Fmsrnn(XSkip)=RNN(Fms(XSkip))(16)=RNN(F5×d(concatd([XSkip,U×2(D×12(XSkip)),U×4(D×14(XSkip))]))).

Here, $U_{\times m}$ and $D_{\times \frac{1}{m}}$ are non-linear upsampling and downsampling operators (factor *m*), respectively. In contrast to previous convolution operations (Section 3.3.1) we employ a convolutional kernel of size k×d to extract temporal-channel representations by convolving along the time axis and with regards to the entire feature channel information. The features from each rescaling operation are then concatenated along the feature channel axis and further processed through the bottleneck layer. The output of that bottleneck layer then serves as input to the RNN layer.

### 3.5. Uncertainty Modeling for Noisy Samples

The proposed model is trained using the large virtual IMU dataset that we extract from YouTube by utilizing the IMUTube system as described in Section 2.3. Such automatically generated training data are likely to contain outliers, i.e., samples of sub-optimal quality as explained below. IMUTube uses search queries as, arguably, weak labels to be associated with the generated virtual IMU data. The videos retrieved from YouTube (or any other public video repository, for that matter) are not guaranteed to only cover the activities that the search query was targeting. Instead, at least parts of the retrieved videos may contain other activities that are irrelevant if not detrimental for our modeling process. Such outlier samples impose, if not treated properly, challenges for HAR models to learn effective motion features for target activities. Previous work showed that handling outlier samples in HAR datasets is important and, if successful, can significantly improve the performance of the resulting HAR model [91,92,93].

To effectively train our HAR model with a dataset that potentially contains “noisy” samples, we integrate a sample uncertainty quantification model that effectively assesses the quality of training samples through their degree of “noise” both at sample- as well as label-level. The model identifies outliers in a dataset, i.e., samples that are deemed of lower quality due to the aforementioned noise. It is based on the principle of heteroscedastic aleatoric uncertainty, which is defined as input-dependent uncertainty for the samples in a given task [94,95]. Kendall et al. [94] quantify sample-specific uncertainties as a function of input, which was predicted along with class labels. The quantified uncertainty of a sample is used to regularize the amount of backpropagated gradient from the classification loss that is actually used for the model parameter update. In doing so, the model selectively learns from those samples in a dataset that are more relevant for deriving effective feature representations for the target activities.

**Figure 7:**
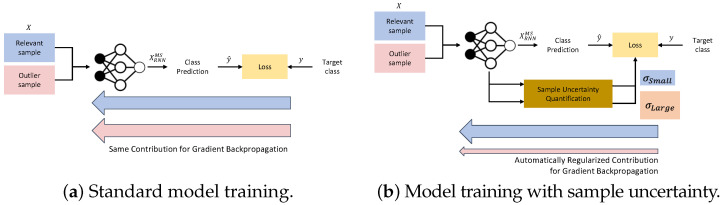
Comparison between model training with (right) and without considering sample uncertainty (left). Information flows related to outlier and relevant samples are colored in red and blue, respectively. The size of the backward arrows illustrates the relative contribution of the samples to the overall gradient signal as it is distributed to the model parameters through backpropagation. (**a**) For standard model training, the contributions from outliers and relevant samples are the same for the model update. (**b**) Our uncertainty assessment model quantifies the degree of uncertainty for a given sample and then regularizes model training accordingly.

Figure 7b illustrates the robust model training procedure that automatically focuses on more relevant training examples. For those samples that the uncertainty assessment procedure determines to be of higher relevance for model training, the relative weight, i.e., their contribution to model training, is increased. In contrast, the weights for outlier samples is decreased. Formally, for a feature vector $x_{RNN}^{MS}=F_{msrnn}\left(X^{Skip}\right)$, extracted by a multi-scale RNN model (Section 3.4.3), the uncertainty learning (UL) model quantifies the sample uncertainty $\sigma =F_{ul}\left(x_{RNN}^{MS}\right)\in R$, which is used to regularize the cross-entropy loss for the overall model $L_{c}\left(W\right)=-\log\;softmax\left(y,\hat{y}=F_{cls}\left(x_{RNN}^{MS}\right)\right)$ measured between target class *y* and the predicted class $\hat{y}$, where $F_{cls}\left(\cdot \right)$ represents the standard softmax classifier for multi-class classification:

(17) $$L\left(W,\sigma \right)=\frac{1}{\sigma_{2}^{2}}L_{c}\left(W\right)+\log\sigma .$$

For $F_{ul}\left(x\right)$, we use two fully-connected layers with ReLU activations. As the uncertainty σ increases, the cross-entropy loss $L_{c}\left(W\right)$ decreases. At the same time, σ is regularized to avoid pushing σ→∞, which would result in effectively ignoring $L_{c}\left(W\right)$ for the entire loss L(W,σ). By using aleatoric uncertainty, the learning signal from noisy samples that do not belong to the target activity is regularized for backpropagation.

To summarize, the proposed model captures the core motion signal of a sensor analysis window and extracts multi-scale, multi-window, and multi-view (time-channel) feature representations. The resulting model is trained end-to-end thereby explicitly incorporating sample uncertainty estimations as effective means for model regularization. The presented modifications and extensions to the HAR model result in an substantial increase in the number of trainable model parameters. As we hypothesized, the increase in model complexity leads to more flexible and robust HAR models—if trained robustly on a suitably sized training dataset. In fact, the proposed model contains approximately 1100 times more trainable model parameters compared to a standard convolutional model. Given that the limitations on the size of a labeled training dataset can be alleviated through the use of the IMUTube system, the novel, complex HAR model can now be trained effectively as will be demonstrated in the next section.

## 4. Case Study: Analyzing Free Weight Gym Exercises

Based on our assumption that more complex activity recognition models lead to improved classification accuracy—if the models can be suitably trained—we now evaluate the novel HAR model architecture on an exemplary, challenging analysis task. Using the IMUTube system, we generated a large dataset of virtual IMU sensor readings, which puts us in the position to be able to train the complex model architecture introduced in Section 3 in a robust manner.

In what follows, we will first introduce the case study and provide details of the dataset generated using IMUTube. We then give details on the experimental evaluation in which we not only compare classification capabilities of the overall, new HAR model architecture to the state-of-the-art in the field, but also analyze the impact each individual component of the new model architecture has on the overall assessment capabilities.

### 4.1. Scenario

For the experimental evaluation of the proposed model, we focus on free-weight gym activity classification where we capture movement data with a single wrist-worn inertial measurement unit. Specifically, we analyze 13 dumbbell exercise classification tasks (Table 1), which were considered the most challenging activities in previous, related studies [69]. Many dumbbell exercises exhibit very similar motions (low inter-class variability), while at the same time substantial intra-class variability can be observed due to differences in individual posture, weight, and skill level.

We investigate how the increase in model complexity impacts activity recognition accuracy when the classifiers can be robustly trained using a large-scale, virtual IMU dataset. As we implement all of the proposed models in Section 3, we will evaluate changes in classification performance as we integrate them one at a time from the standard convolutional neural network model. Furthermore, incrementally adding each module allows us to analyze the effectiveness of the proposed models individually, which will provide suggestions for further in-depth investigation in future research.

### 4.2. Datasets

The IMUTube system requires small portions of real IMU data to be used along with the virtual IMU data as they are produced by the framework [14]. These real sensor samples are used for calibration, i.e., to match the generated virtual IMU data with the real life scenarios. As shown in the original IMUTube publications, the amount of real sensor data is very small indeed, amounting to only a fraction of the virtual IMU data. Replicating previous protocols ([14,15]), we use the MyoGym dataset [69] as a source for real sensor data to be used in our experiments. It includes 30 free-weight activities from 10 users. On average, we have 2.81 min of real IMU data available per activity.

For virtual IMU data, we generated a large dataset through using IMUTube as is and with the names of the 13 selected dumnbell exercises as search terms in YouTube. The resulting dataset contains approximately 3.20 h per activity (>41 h of virtual data total) with YouTube search terms serving as (weak) labels [15].

### 4.3. Evaluation Protocol

Typical YouTube videos are recorded with a 30-Hz frame rate. In order to match the characteristics of real and virtual IMU data, we downsampled the real IMU data to 30 Hz (from the original 50 Hz). For the sensor frame size input to the model, we use a 4-second window with 1-second overlap across all experiments, as a single repetition of concentric and eccentric contractions is known to take between 3 to 4 seconds [78].

For the MyoGym real IMU dataset, we use subjects 1 through 5 as training set, subjects 6 and 7 as validation set, and data from subjects 8 through 10 as the test set for all experiments. For evaluation metrics, the mean F1 score is used to account for label imbalance in the dataset. For the statistical significance test, Wilson score intervals are derived from 15 runs for each experiment [96].

The use of virtual IMU data for HAR model training requires calibration with small amounts of real IMU data to resolve domain discrepancies between the real and virtual IMU datasets [14]. To do so, we replicate the training protocol from [15] and train the proposed HAR model with both real and virtual IMU data. We first calibrate the virtual IMU dataset with the training set from the real IMU dataset. Next, the model is pretrained with the calibrated virtual IMU data and then fine-tuned with training sets in the real IMU dataset. Testing is based on the model with the highest validation score in the real IMU dataset.

### 4.4. Model Hyperparameters

For model training, regardless of using real or virtual IMU data, the batch size is fixed to 16. The learning rate is fixed to $1\times 10^{-3}$ with the Adam optimizer. When fine-tuning with real IMU data for the model pretrained with virtual IMU data, we reinitialize the last classifier layer and set the learning rate to $1\times 10^{-5}$. Unless specified differently, we set the number of feature maps to 64 and kernel size to 5×1 for all convolutional modules in our model and use two layers of fully connected layers with 128 units for the final classification layers, which is in line with related work [7]. Furthermore, except for the first layer with raw sensor input, max-pooling operation with a 2×1 kernel with 2×1 stride is used to downsample the size of feature resolution at each layer.

### 4.5. Results

Table 2 lists the evaluation results for the comparison of the effectiveness of the proposed model architecture to the state-of-the-art. As baselines, we compared the proposed method with a four-layer convolutional neural network (ConvNet) and the DeepConvLSTM architecture [7]. For the proposed model, we incrementally applied the individual modules as they were introduced in Section 3 to explore not only the overall effectiveness of the new model architecture but also the impact each part has on the recognition accuracy. By doing so, we can assess the increase in classification performance in relation to the increasing number of trainable parameters, which represents the model complexity. When trained with the virtual IMU dataset, our best model (6th row in the “proposed model” block in Table 2, AT + MS + MW + MV + AX + RNN) achieves an 80.2% mean F1 score, which is a significant improvement over the state-of-the-art baselines: +17.6% absolute when comparing to the CNN baseline, and +6.2% absolute when comparing to DeepConvLSTM. It is worth reiterating that these substantial gains in recognition accuracy come with no additional costs with regard to collecting and annotating training data but can exclusively be attributed to the fact that with the availability of high-quality virtual IMU data, we can now train more complex HAR models in a robust manner.

Table 2 documents the changes in recognition accuracy (mean F1 scores) aligned with the increasing model complexity, indicated through the number of trainable model parameters. Using all proposed modules (last row), the final model architecture contains ca. 1100× more trainable model parameters than the convolutional modeling baseline (Convnet). The corresponding gain in recognition accuracy totals to a 12.64% absolute F1 score when using real IMU, and a 17.63% absolute F1 score when using both real and virtual IMU data. The performance gain when using virtual IMU dataset was more pronounced for the more complex model (as directly listed in the last column—Δ).

As soon as adaptive trimming (AT) is introduced (Table 2, 1st row under “Proposed”), the number of model parameters grows by a factor of about 10 when compared to ConvNet. This demonstrates that detecting core motions before the actual feature extraction step helps improve model performance significantly, resulting in an average gain of 4.33% F1 score for both real, and real+virtual IMU data scenarios.

The introduction of multi-window size kernels (MW-Conv) increases the model complexity by another factor of 10 (second row under “Proposed” in the table). To extract multi-scale feature representation at each layer, we applied non-linear rescaling with factors of $\left[1,\frac{1}{2},\frac{1}{4},\frac{1}{8}\right]$ (Section 3.3.1). Capturing multi-scale features was very effective for increasing model performance in a statistically significant manner with an increase of 3.44% in the absolute F1 score on average.

The addition of multi-view kernels (MV-Conv) increased the model complexity by another factor of 4 (Table 2, third row under “Proposed”). Capturing varying lengths of motion signal significantly improved the model performance, resulting in an average F1 score gain of 3.58% absolute for both real, and real+virtual IMU training datasets. To capture varying durations for activities under study, we employed 10 different 1D kernel sizes, namely [3,5,7,15,23,31,37,45,53,61]. For a 30-Hz signal, each kernel size corresponds to 0.125 s, 0.166 s, 0.25 s, 0.5 s, 0.75 s, 1 s, 1.25 s, 1.5 s, 1.75 s, and 2 s of sensor data, respectively. Observing the effectiveness of using an extremely large kernel window size over 1-second calls for further investigation on the use of large kernel windows specifically for modeling a sensor time-series.

Modeling time-channel representations with varying kernel shapes (fourth row under “Proposed” in the table) improved model performance by 1.15% for absolute F1 score on average for both training datasets, which represents a statistically insignificant improvement. The model trained only with real IMU data showed only a marginal gain, namely an increase of 0.45% for the F1 score absolute. We consider that a single wrist sensor with three channels did not provide many benefits from modeling the channel axis explicitly. Modeling a single wrist sensor depends more on how to encode temporal information, as we could observe from the analysis in multi-scale multi-window convolution.

Introducing skip-connections into the model increased the overall complexity of our model to 402 times the complexity of the reference ConvNet. The resulting recognition accuracy increased by 2.65% for the F1 score absolute when training with virtual IMU data and 0.32% in the F1 score when only using real IMU data (Table 2, fifth row under “Proposed”). This shows that skip-connections help to avoid the vanishing gradient effect in the bottom layers and that these auxiliary connections have a bigger positive impact when variable gradient signals are available from large-scale virtual IMU datasets.

Introducing recurrency into the model (sixth row under “Proposed”) resulted in marginal improvements in model performance, namely an absolute 0.19% F1 score increase when using both virtual and real IMU data for training. In this experiment, we used a two-layer GRU model with 1536 units for each layer. As discussed in Section 3.4.3, we applied non-linear multi-scale operation on the input at each layer of GRU with scaling factors of $\left[1,\frac{1}{2},\frac{1}{4}\right]$. We also explored different sizes of GRU units, different numbers of layers, and replacing GRU with LSTM, but neither of them provided statistically significant differences. We consider that aggregating temporal information has an insignificant effect due to the usage of multi-scale multi-window kernel convolution. The convolutional layer already captures long-range motion signal from using the kernel that covers two seconds of data in a four-second analysis window. In addition, the multi-scale operation enables the model to capture long-term temporal dynamics at multiple scales.

The effect of using uncertainty modeling (Table 2, last row under “Proposed”) for pretraining was marginal not resulting in significant performance differences (0.27% absolute F1 score increase). This shows that, when sufficiently large training data is provided, the complex model can learn effective feature representations that are already robust to label noises, which is in line with previous studies [97]. As discussed in Section 3.5, the feature representation from the last layer of the GRU model is forwarded to the classifier and uncertainty detection model. In this experiment, we used a two-layer fully-connected model with ReLU activation. To gradually decrease the feature dimension from the GRU layer (1536) to a single real value regression for quantifying uncertainty, we used 768 and 384 units for each layer.

## 5. Discussion

In this paper we have explored how modeling for human activity recognition using wearable movement sensors can be changed if the typical restriction of not having sufficient amounts of labeled training data effectively disappears. Building our work on systems like IMUTube allowed us to focus our efforts on capturing the relevant essentials of IMU data without being constrained by keeping the number of trainable parameters low for robust model training. We have successfully demonstrated how more complex models lead to substantial improvements in HAR accuracy, given that we are now in the position to train these in a suitable way. The large amount of easy-to-retrieve virtual IMU data is key to this paradigm change.

Our work opens up opportunities for future work in this field. In what follows we discuss potential next steps along with a general call for further exploration and concerted developments across communities. We also show and discuss limitations of our approach in its current form.

### 5.1. Collect Even Larger Datasets of Virtual IMU Data

The substantial improvements in HAR accuracy, which we have shown in this paper, indicate that it is possible to derive more complex HAR models using virtual IMU data and that the increased complexity, in terms of number of model parameters that can be trained *robustly*, is, in fact, the reason for the performance improvements. The dataset that we used in this paper was generated by using the IMUTube system on an exemplary, challenging recognition task, namely, the assessment of free-weight gym exercises. Compared to the original dataset that contains real IMU data, we could increase the amount of (weakly) labeled training data by a factor of 100 to a total of approximately 41 hours, i.e., about 150,000 samples of 4-second data frames, which are the basis for our analysis.

Even though this increase in training data is substantial, the dataset we used is still relatively small when compared to datasets as they are typically used in other analysis domains that utilize machine learning models. For example, the popular ImageNet dataset consists of about 14 million annotated images [98], which is two orders of magnitude larger than our dataset. With such a massive dataset it is now possible to train very large models, such as the popular AlexNet [99], or ResNet-152 [86] that consists of 60 million trainable parameters. Encouraged by the promising results presented in this paper, we hypothesize that generating even larger datasets of virtual IMU data will further improve recognition accuracy in supervised HAR scenarios. Yet, it remains to be explored to what extent the inevitable increase of noise that is introduced into the virtual IMU dataset will counteract or perhaps even eliminate the performance gains that can be made by increasing the size of the training datasets. In contrast to the aforementioned examples from the image processing/computer vision community, the additional training data are *virtual* IMU data and not real sensor readings.

### 5.2. Analyze Complex Activities

While assessing the free-weight exercises is a challenging task, the underlying movements are relatively constrained. Many physical activities are either more complex or more subtle (or both) than the exemplary gym exercises that we studied in this paper. As such, future studies should expand towards other, complex activities to explore how virtual IMU data can effectively be utilized for improved modeling in HAR. Recently, Liu et al. [16] demonstrated that virtual IMU data from videos could be used for American Sign Language (ASL) recognition tasks. Signs in ASL, arguably, resemble complex activities where subtle changes often result in entirely different semantics of the underlying movement. The reported preliminary results indicate that systems like IMUTube can be used to successfully generate training data for more complex activities than the ones studied in this paper.

### 5.3. End-to-End Learning of Complex Model Architectures

We designed the complex model architecture presented in this paper with the specific focus on capturing relevant aspects of the underlying movement data as they are of importance for human activity recognition using body-worn movements sensors. The result of these design efforts is a complex model architecture (Section 3) that includes a large number of individual, manually-defined components. While the design of these components, and hence the analysis model overall, was done manually by experts who have been working in the field for many years, the model parameters itself were learned automatically from virtual training data.

While the achieved performance improvements are substantial, which confirms our assumption that more complex models lead to improved classification performance if the models can be trained suitably, it, arguably, begs the question if those model extensions itself could have been learned automatically from the training data. The emerging field of Neural Architecture Search (NAS [100]) studies how model topologies–rather than model parameters only–can be learned automatically. For example, one could include the number of hidden layers of a network, or the connectivity between layers (to name but a few examples) into the learning process. NAS methods typically employ meta-learning, such as genetic algorithms or reinforcement learning–each based on specific utility functions–to automatically derive model topologies/architectures. It remains to be seen if such fully automated, end-to-end learning approaches would lead to similar or even further improvements in classification accuracy.

### 5.4. Virtual IMU Data as Basis for Alternatives to Supervised Learning

The main motivation for systems like IMUTube is to overcome the shortage of *labeled* training data in machine learning-based HAR scenarios. The work presented in this paper essentially falls into the same category of supervised training of, now more complex, machine learning models. While it is encouraging to see the substantial improvements in classification accuracy in HAR through the use of large amounts of weakly labeled virtual IMU data, it is worth expanding the view towards alternatives to conventional, supervised learning approaches.

Recently, semi-supervised and especially self-supervised learning methods [101,102,103] have become popular in many machine learning application domains including human activity recognition using wearables [13,43,44]. Here the idea is to enhance small amounts of labeled data through specific modification, formalized through so-called pretext learning tasks, such that, through solving the auxiliary task, a meta-learning procedure is forced to learn higher-level concepts that eventually lead to improved activity recognition performance. Future studies could explore to what extent large scale virtual IMU dataset can be utilized to support self-supervised learning methods.

Relaxing the requirements on annotations even further, one could explore to what extent virtual IMU data can be used for fully unsupervised learning scenarios where, for examples, feature representations are learned directly from raw sensor data [6,24].

## 6. Conclusions

With the development of systems like IMUTube [14], it has now become possible to generate virtually unlimited amounts of weakly labeled, virtual IMU data. As such, modeling for human activity recognition using wearables (HAR) is, in principle, no longer constrained to account for the typically rather small labeled sample sets. In this paper, we utilized a large, virtual IMU dataset to develop complex HAR models that include substantially larger amounts of trainable model parameters than state-of-the-art models in the field. Our assumption was that more complex models lead to improved recognition performance if the models can be trained sufficiently.

We presented a HAR model that contains more than 1100 times more trainable parameters than state-of-the-art models. Using a dataset that contains 41 hours of virtual IMU data and a small amount of real IMU data for calibration, we were able to train the new model robustly and could demonstrate substantial improvements in classification accuracy on an exemplary, challenging assessment task, namely the analysis of free-weight gym exercises captured by wrist-worn inertial measurement units. Our findings are significant because they show that more complex models indeed lead to an improved HAR performance, and also that such more complex models can actually be derived in a robust manner by utilizing virtual IMU data that can easily be generated using systems such as IMUTube.

## Figures and Tables

**Table 1:** A total of 13 dumbbell activities from MyoGym [69] datasets for evaluation.

Name	Muscle Group	Posture	One-Arm, Both or Alternate
One-Arm Dumbbell Row	Middle Back	Bent Over	One-arm
Incline Dumbbell Flyes	Chest	Seated inclined	Both
Incline Dumbbell Press	Chest	Seated inclined	Both
Dumbbell Flyes	Chest	On back	Both
Tricep Dumbbell Kickback	Triceps	Bent Over	One-arm
Dumbbell Alternate Bicep Curl	Biceps	Standing	Alternate
Incline Hammer Curl	Biceps	Seated inclined	Both
Concentration Curl	Biceps	Seated	One-arm
Hammer Curl	Biceps	Standing	Alternate
Side Lateral Raise	Shoulders	Standing	Both
Front Dumbbell Raise	Shoulders	Standing	Alternate
Seated Dumbbell Shoulder Press	Shoulders	Seated	Both
Lying Rear Delt Raise	Shoulders	On stomach	Both

**Table 2:** Classification performance (mean F1 score, Wilson score interval) for our experimental evaluation. The baseline models are a standard four-layer convolutional models (ConvNet) and the DeepConvLSTM architecture [7]. Starting from ConvNet, we incrementally added individual components of the proposed model architecture to evaluate the effect of each of them along with the assessment of the overall effect more complex HAR model architectures have on recognition performance. The components are listed as follows: Adaptive trimming (AT, Section 3.2), multi-scale feature extraction (MS, Section 3.3.1), multi-kernel window feature extraction (MW, Section 3.3.2), multi-view convolution for temporal-channel feature extraction (MV, Section 3.3.3), skip connections from intermediate layer features (AX, Section 3.4.2), 2-layer MS-GRU unit for temporal aggregation of convolutional features (RNN, Section 3.4.3), and sample uncertainty modeling (UL, Section 3.5). The number of model parameters is shown to provide a direct comparison of the model complexity. The last column shows the performance difference, Δ in percentage, when using virtual IMU data for model training.

Model							Number of Parameters	Training Data		Δ
								Real IMU	Real + Virtual IMU	
ConvNet							106,054	0.589±0.026	0.625±0.026	5.7%
DeepConvLSTM							394,189	0.594±0.025	0.740±0.023	19.7%
Proposed										
AT	MS	MW	MV	AX	RNN	UL				
✔	✕	✕	✕	✕	✕	✕	1,239,519	0.622±0.026	0.679±0.026	8.3%
✔	✔	✕	✕	✕	✕	✕	1,335,519	0.656±0.026	0.714±0.024	8.1%
✔	✔	✔	✕	✕	✕	✕	10,855,199	0.687±0.025	0.755±0.023	9.0%
✔	✔	✔	✔	✕	✕	✕	42,727,455	0.691±0.025	0.774±0.022	10.6%
✔	✔	✔	✔	✔	✕	✕	42,933,599	0.695±0.025	0.800±0.021	13.1%
✔	✔	✔	✔	✔	✔	✕	112,810,015	0.707±0.024	0.802±0.021	11.8%
✔	✔	✔	✔	✔	✔	✔	116,473,632	0.716±0.024	0.799±0.021	10.4%
